# Supplementary figures and images for: Multi-task transfer learning for the prediction of entity modifiers in clinical text: application to opioid use disorder case detection
Source: J Biomed Semantics. 2024 Jun 7;15:11. doi: 10.1186/s13326-024-00311-4 (PMC11157899; doi:10.1186/s13326-024-00311-4)

**Supplementary Materials**


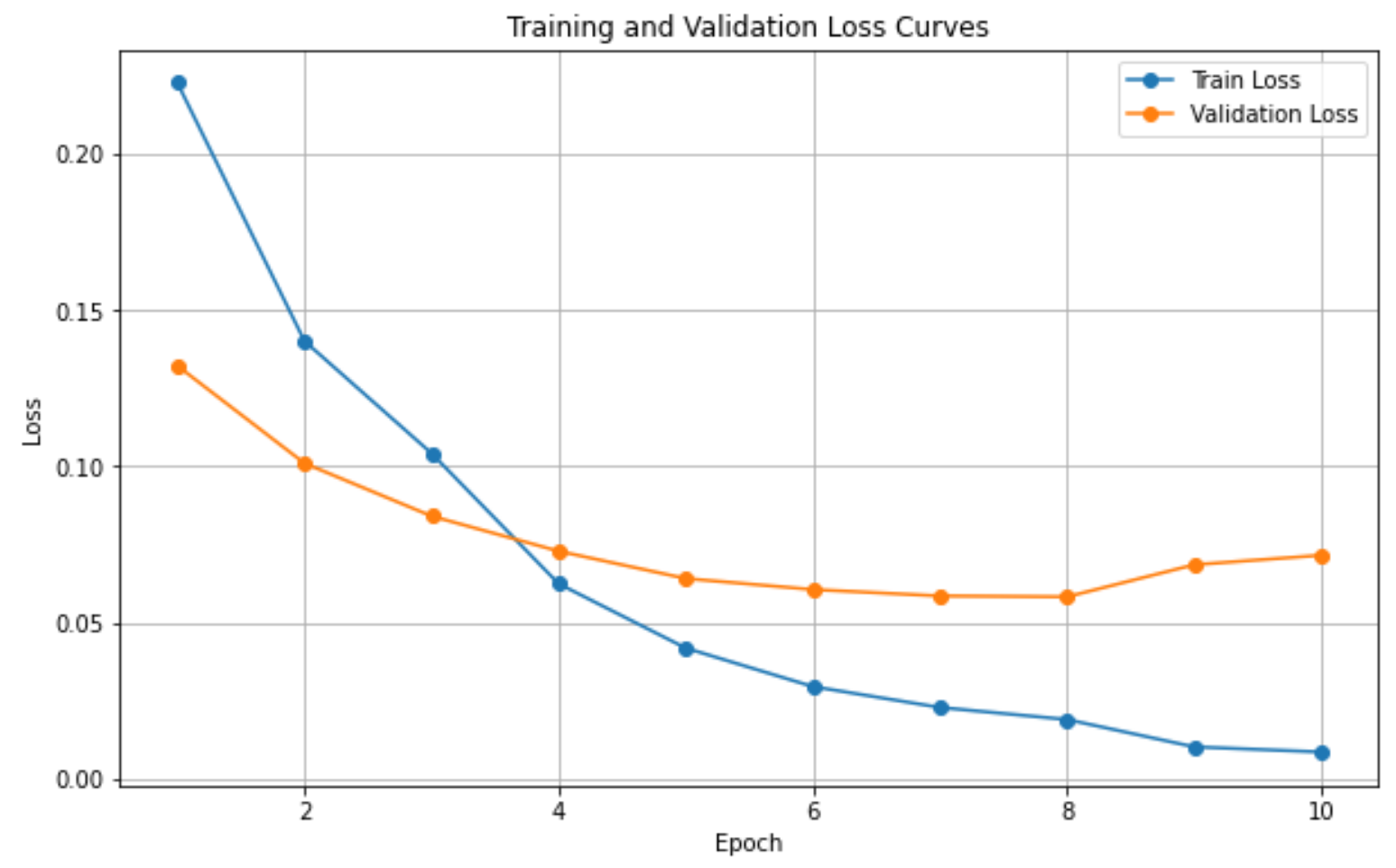


Figure 1. Training and validation losses for MT-SHR.

Supplement: Supplementary file 1 — Supplementary Material 1. [file 13326_2024_311_MOESM1_ESM.docx]
